# Educational gradients in the quality of mortality data: a nationwide, registry-based study on heart failure listed incorrectly as underlying cause of death in Norway

**DOI:** 10.1177/14034948241296239

**Published:** 2024-11-24

**Authors:** Gerhard Sulo, Ann Kristin Knudsen, Carl Baravelli, Christian Lycke Ellingsen, Enxhela Sulo

**Affiliations:** 1 Department of Global Public Health and Primary Care, University of Bergen, Norway; 2 Department for Disease Burden, Division of Mental and Physical Health, Norwegian Institute of Public Health, Bergen, Norway; 3 Department of Pathology, Stavanger University Hospital, Norway; 4 Department of Heart Disease, Haukeland University Hospital, Bergen, Norway

**Keywords:** Heart failure, garbage codes, mortality, education, Norway, epidemiology

## Abstract

**Aim:**

In the context of mortality, heart failure (HF) represents an intermediate factor and should not be used to describe underlying cause of death (UCoD). We explored the potential educational gradients in use of HF to describe UCoD using national data spanning more than 30 years from Norway.

**Methods:**

Using a cross-sectional design, we linked data from the Cause of Death Registry and the National Education Database. Logistic regression models were used to analyze the association between highest attained education and the odds of HF being listed as the UCoD: odds ratios (ORs) and corresponding 95% confidence intervals (CIs) are reported.

**Results:**

HF was listed as UCoD in 46,331 (3.7%) of 1,254,249 deaths analyzed. Compared to primary education, secondary and tertiary education were associated with 10% (OR = 0.90, 95% CI: 0.88–0.92) and 17% (OR = 0.83, 95% CI: 0.80-0.86) lower odds of HF incorrectly listed as UCoD, respectively. We observed no significant differences for the association between education and study outcomes between men and women and across place of death categories. However, educational gradients were greater among younger compared to older individuals (*p*_interaction_, = 0.002). Similar educational gradients were observed in the analyses restricted to cardiovascular deaths (OR = 0.93; 95% CI: 0.91–0.94 for secondary vs. primary education, and OR = 0.91; 95% CI: 0.88–0.95 for tertiary vs. primary education).

**Conclusions:**

**Education was inversely associated with the use of HF to incorrectly describe UCoD. Addressing the observed educational gradients, would improve the quality of mortality data and allow for less biased descriptions of cause-specific mortality.**

## Introduction

Cause-specific mortality data are crucial for understanding the main health drivers and their related risk factors in communities. Using the information available on death certificates and applying a strict set of rules described by World Health Organization (WHO), each death is assigned an underlying cause. The underlying cause of death (UCoD) is defined as “the disease or injury initiating the train of morbid events leading directly to death” (WHO, 1994). As such, it must be unique and exhaustively descriptive of the condition leading to death. However, the process of identifying the correct UCoD can often be challenging. Many deaths are ascribed to “garbage codes” (i.e., codes denoting conditions that are not specific enough, or are an immediate, intermediate, and even impossible causes of death) [1]. Extensive use of garbage codes to describe UCoD is an important indicator of poor-quality mortality data and can distort the true picture of cause-specific mortality [2].

Heart failure (HF) represents the end stage of many chronic conditions and is a common reason for hospitalization. However, in the context of mortality, it is only a mediator between the true underlying condition, and death. Thus, it should not be listed as the UCoD. Yet, HF represents the most frequently used garbage code within the cardiovascular disease (CVD) chapter of the International Classification of Diseases (10^th^ revision; ICD-10). Studies have shown that replacement of HF with the true UCoD results in important changes in mortality classifications, from conditions such as ischemic heart disease, hypertensive heart diseases, cerebrovascular diseases, diabetes mellitus, and lower respiratory infections [3–5]. Therefore, extensive use of HF to describe UCoD not only has a negative impact on the quality of vital statistics but can also bias the true picture of cause-specific mortality.

Education is a reliable indicator of socioeconomic status (SES). Many studies have reported educational gradients in the incidence, prevalence, and outcomes of various health conditions [6–9]. A handful of international studies have pointed to another, less discussed field, displaying SES gradients, namely, the quality of vital statistics [10–12]. Taken together, these studies indicate that there might be a link between SES and the quality of mortality data. However, the generalizability of these studies is limited by their small sample sizes and an ecological study design [10–12].

Using individual-level, national data spanning a more than 30-year period in Norway, the aim of the current study was to investigate the potential association between education—a reliable indicator of SES—and the use of HF to describe the UCoD.

## Materials and methods

### Data sources, study exposure, and outcome

Information from the Norwegian Cause of Death Registry (NCDR) [13], and the National Education Database (NEDB) were linked at the individual level, using a project-specific unique identifier. The NCDR contains information on sex, date, age and place of death, underlying and contributing causes of death, and whether an autopsy (forensic or medical) was performed. The NEDB contains information on the highest achieved education for all persons with a permanent address in Norway. It is based on reports from educational institutions, which are submitted to Statistics Norway and is updated yearly. The highest achieved education is coded according to the Norwegian standard classification. For these analyses, we classified education level (i.e., study exposure) into three categories: primary (up to 10-years’ compulsory education), secondary (high school or vocational school), and tertiary education (college/university).

For all deaths, we scrutinized the provided codes listed as UCoD and deceased for whom an HF code (ICD-9 codes: 416–416.9, 428–428.9, and ICD-10 codes: I27.8, I27.9, I50–I50.9, J81, J81.1) was listed as UCoD (study outcome).

### Target codes

In addition to UCoD, we scrutinized all additional codes used to describe intermediate, immediate (included in Part I of the death certificate), and contributing (included in Part II of the death certificate) causes of death. Based on this additional information, we categorized individuals into those with, and those without a “target code” in either Parts I or II of their death certificate. The term target code refers to the medical conditions to which “HF deaths” (i.e., deaths with HF listed incorrectly as UCoD) should in principle be reassigned to, based on the pathophysiology or an assessment of certification practices [14]. A list of target codes for HF is provided by Johnson et al. [1] and the corresponding conditions are included in the footnote of Table V. This was done to explore how other relevant information, which could have been used to correctly identify the true UCoD, was distributed across the educational strata.

### Education level and HF listed as UCoD

Logistic regression models were used to estimate the odds ratios (ORs) and 95% confidence intervals (CIs) for the association between education level and HF listed as UCoD. Analyses were adjusted for sex, age at death (in 5-year strata), year of death (Model 1) and, additionally, for autopsy performed upon death and place of death (Model 2)—all variables that have been shown to independently predict the use of garbage codes [15] and associated with education level in our data.

For the period 1984–2004, the NCDR did not contain specific information on immediate, intermediate, or contributing causes of death. Such information became available from 2005 onward. To account for this historical transition in the NCDR’s structure, we additionally stratified the analyses on study period (1986–2004 and 2005–2015).

In most cases with HF listed (incorrectly) as UCoD, the true cause of death would have been a CVD condition [1]. Hence, we repeated the analyses restricting the study population to those dying from CVD (ICD-9 codes: 390–459; ICD-10 codes: I00–I99).

Lastly, we tested whether the magnitude and strength of the association being studied was different between men and women and across age at death and place of death strata by including interaction terms (in separate models) between education and (i) sex, (ii) place of death, and (iii) age at death. After that, we conducted stratified analyses by sex, place of death and age at death and summarized the results in Table IV.

The study is part of a project funded by the Research Council of Norway (Norwegian Burden of Disease Study 2017–2019: regional and socioeconomic patterns in Norway and Nordic comparison studies) and was approved by the Regional Committee for Medical and Health Research Ethics South-East Norway (2013/2394). All analyses were performed using STATA software version 16.

## Results

HF was incorrectly listed as UCoD in 46,331 (3.7%) of all deaths occurring in Norway during the period 1986–2015 (Table I). The corresponding proportions across education categories were 4.1%, 3.2%, and 2.7% for primary, secondary, and tertiary education, respectively. Individuals with tertiary education were predominantly men, died in hospital more often, and more frequently underwent an autopsy compared to individuals with primary education. We observed no educational differences regarding age at death among the deceased with HF listed as UCoD.

**Table I.** Characteristics of deaths (total and those with heart failure listed incorrectly as underlying cause of death) by education level in Norway, 1986–2015.

Characteristics of study participants	All deaths (*n* = 1,254,249)			Deaths with HF listed incorrectly as UCoD (*n* = 46,331)		
	Primary (*n* = 705,099)	Secondary (*n* = 445,171)	Tertiary (*n* = 102,954)	Primary* (*n* = 29,193)	Secondary** (*n* = 14,368)	Tertiary (*n* = 2770)
Sex, %						
Men	44.4	54.6	61.8	34.5	43.9	53.5
Women	55.6	45.4	38.2	65.5	56.1	46.5
Age at death, median (IQR)	81 (73–87)	79 (69–86)	77 (65–86)	87 (82–91)	86 (81–91)	87 (82–91)
Place of death, %						
Hospital	38.1	41.5	43.4	19.6	22.2	25.6
Nursing home	42.4	36.5	32.3	64.2	60.6	56.5
Home	15.4	16.7	17.4	13.9	14.6	14.5
Other	4.1	5.3	6.9	2.3	2.6	3.4
Autopsy, %						
No	91.7	89.6	87.0	99.4	99.2	98.8
Yes	8.3	10.4	13.0	0.6	0.8	1.2

*Note*: UCoD: underlying cause of death; IQR: interquartile range; HF: heart failure.

Primary: up to 10 years’ compulsory education; secondary: high school or vocational school; tertiary: college/university.

### Education level and HF listed as UCoD

Over the whole study period, secondary and tertiary education were associated with 10% (OR = 0.90; 95% CI: 0.88–0.92) and 17% (OR = 0.83; 95% CI: 0.80–0.86) lower odds of HF being listed as UCoD compared to primary education (Table II).

**Table II.** Association between education level and use of heart failure listed incorrectly as underlying cause of death in Norway: overall (1986–2015) and by two study periods (1886–2004 and 2005–2015).

Education	Odds ratio (95% confidence interval)	
	Model 1^*^	Model 2^**^
	Period 1986–2015 (*n* = 1,254,249)	
Primary	1 ^ref^	1 ^ref^
Secondary	0.89 (0.87–0.91)	0.90 (0.88–0.92)
Tertiary	0.81 (0.77–0.84)	0.83 (0.80–0.86)
	Period 1986–2004 (*n* = 816,180)	
Primary	1 ^ref^	1 ^ref^
Secondary	0.88 (0.86–0.91)	0.90 (0.88–0.93)
Tertiary	0.77 (0.73–0.81)	0.80 (0.76–0.85)
	Period 2005–2015 (*n* = 438,069)	
Primary	1 ^ref^	1 ^ref^
Secondary	0.90 (0.87–0.94)	0.91 (0.88–0.94)
Tertiary	0.86 (0.81–0.92)	0.87 (0.82–0.93)

* Adjusted for age at death, sex, and calendar year.

** Adjusted for age at death, sex, calendar year, place of death, and autopsy performed upon death.

Primary: up to 10 years’ compulsory education; secondary: high school or vocational school; tertiary: college/university.

When stratifying the analyses by study period (1986–2004 and 2005–2015), the strength of the association between education and HF listed as UCoD did not change for secondary (vs. primary) education but weakened slightly for tertiary (vs. primary) education (for the period 1986–2004: OR = 0.80; 95% CI: 0.76–0.85; and for the period 2005–2015: OR = 0.87; 95% CI: 0.82–0.93) (Table II).

When analyses were restricted to deaths within CVD chapter (*n* = 514,113), the association between education and HF listed as UCoD still persisted although the strength of the association was attenuated compared to the overall analyses (OR = 0.93; 95% CI: 0.91–0.94 for secondary vs. primary education; and OR = 0.91; 95% CI: 0.88–0.95 for tertiary vs. primary education) (Table III).

**Table III.** Association between education level and heart failure listed incorrectly as underlying cause of death in Norway: analyses restricted to 514,113 deaths within cardiovascular diseases chapter.

Education	Odds ratio (95% confidence interval)	
	Model 1^*^	Model 2^**^
Primary	1 ^ref^	1 ^ref^
Secondary	0.92 (0.90–0.94)	0.93 (0.91–0.94)
Tertiary	0.87 (0.83–0.91)	0.91 (0.88–0.95)

* Adjusted for age at death, sex, and calendar year.

** Adjusted for age at death, sex, calendar year, place of death, and autopsy performed upon death.

Primary: up to 10 years’ compulsory education; secondary: high school or vocational school; tertiary: college/university.

Educational gradients in HF listed as UCoD were comparable between men and women (tertiary vs. primary education: among men, OR = 0.83; 95% CI: 0.79–0.88; and among women, OR = 0.82; 95% CI: 0.78–0.87; *p*_interaction_ = 0.304) and across place of death (tertiary vs. primary education: deaths at a hospital, OR = 0.85; 95% CI: 0.78–0.93; deaths at nursing homes, OR = 0.83; 95% CI: 0.78–0.87; deaths at home, OR = 0.78; 95% CI: 0.70–0.87; *p*_interaction_ = 0.605). We did, however, observe that the strength of association between education and HF listed as UCoD was greater among younger compared to older individuals (tertiary vs. primary education: 25–69 years, OR = 0.70; 95% CI: 0.59–0.83; 80–89 years, OR = 0.84; 95% CI: 0.79–0.89; and 90+ years, OR = 0.93; 95% CI: 0.87–0.99; *p*_interaction_ = 0.002) (Table IV).

**Table IV.** Association between education level and heart failure listed incorrectly as underlying cause of death in Norway: analyses are stratified by sex, place of death, and age at death.

Characteristics	Odds ratio (95% confidence interval)		
	Education level		
	Primary	Secondary	Tertiary
Sex			
Men (*n* = 620,657)	1 ^ref^	0.91 (0.88–0.94)	0.83 (0.79–0.88)
Women (*n* = 633,592)	1 ^ref^	0.90 (0.87–0.92)	0.82 (0.78–0.87)
Place of death			
In a hospital (*n* = 498,163)	1 ^ref^	0.88 (0.84–0.92)	0.85 (0.78–0.93)
In nursing homes (*n* = 498,163)	1 ^ref^	0.92 (0.89–0.94)	0.83 (0.78–0.87)
At home (*n* = 498,163)	1 ^ref^	0.89 (0.85–0.94)	0.78 (0.70–0.87)
Age (group) at death			
25–69 years (*n* = 274,952)	1 ^ref^	0.83 (0.75–0.92)	0.70 (0.59–0.83)
70–79 years (*n* = 315,816)	1 ^ref^	0.86 (0.81–0.90)	0.68 (0.61–0.76)
80–89 years (*n* = 462,948)	1 ^ref^	0.90 (0.88–0.93)	0.84 (0.79–0.89)
90+ years (*n* = 200,533)	1 ^ref^	0.94 (0.91–0.98)	0.93 (0.87–0.99)

Primary: up to 10 years’ compulsory education; secondary: high school or vocational school; tertiary: college/university.

Model is adjusted for sex, age at death, year of death, place of death, and autopsy performed upon death.

**Table V.** Proportion of target codes on death certificates of 16,096 deceased with heart failure listed incorrectly as underlying cause of death, 2005–2015.

	Primary (*n* = 8905)	Secondary (*n* = 5859)	Tertiary (*n* = 1332)
	Proportion of target codes in Part I^*^ of death certificate, %		
All deaths	9.4	10.2	10.2
By sex			
Men	11.4	12.3	13.1
Women	8.5	8.5	6.4
	Proportion of target codes anywhere in death certificate, %		
All deaths	22.6	23.7	24.6
By sex			
Men	29.8	29.5	30.4
Women	19.2	19.3	16.6

Target codes for heart failure include congenital heart anomalies, cardiomyopathies, rheumatic heart disease, nonrheumatic valvular heart disease, aortic aneurism, chronic kidney disease, endocrine, metabolic, blood, and immune disorders, endocarditis, ischemic heart disease, cirrhosis and other chronic liver diseases, hypertensive heart disease, chronic obstructive pulmonary disease, diabetes mellitus, tracheal, bronchus, and lung cancer, colon and rectum cancer, intracerebral hemorrhage.

* Listed as intermediate or immediate cause of death in Part I of the death certificate.

Primary: up to 10 years’ compulsory education; secondary: high school or vocational school; tertiary: college/university.

### Relevant information from death certificates of deceased with HF listed as UCoD

Overall, the proportion of individuals for whom we found a target code was low and proportionally distributed across education categories (in Part I of the death certificate: 9.4%, 10.2%, and 10.2% for primary, secondary, and tertiary education, respectively; anywhere in the death certificate: 22.6%, 23.7%, and 24.6% for primary, secondary, and tertiary education, respectively). We observed no educational differences in the quality of additional information (i.e., presence of target codes) after accounting for educational differences with regard to sex and age at death (data not shown).

## Discussion

Our study documented for the first time the presence of an inverse, dose-response association between education and incorrect use of HF to describe UCoD. The association was stronger among younger compared to older individuals but did not differ between men and women or across place of death stratum. The educational gradients in HF listed as UCoD persisted when the analyses were restricted to CVD deaths.

### Comparison with other studies

Three regional ecological studies have addressed the potential association between SES and the quality of mortality data, as measured by the use of garbage codes. The first study was conducted in Amsterdam and reported more frequent use of ill-defined codes in low-income boroughs [10]; the other two studies were conducted in Brazil and reported an association between use of garbage codes and geographical regions’ gross domestic product per capita (a proxy of wealth), Gini index (a proxy of inequalities in outcome) [11], and socio-demographic index: a composite measure capturing fertility, educational attainment, and income per capita [12]. To the best of our knowledge, only one study has explored the association between education level and use of garbage codes using individual-level data. The authors of that study focused on garbage codes included in the “Symptoms, signs and abnormal clinical and laboratory findings, not elsewhere classified” chapter of ICD-10 (otherwise known as “R chapter”) among those deceased at ages 30–79 years across 16 countries [16]. Overall, the use of garbage codes was higher among individuals with low education compared to more affluent people. However, in countries with lower mortality rates, the direction of the association was inverted (i.e., higher use of garbage codes was observed among individuals with higher education). As the analyses were only adjusted for age, it was difficult to establish whether the discrepancies in the direction of association between education and garbage code use was due to residual confounding or reflected differences between countries regarding educational systems, coding practices, and other methodological issues.

### Possible explanations and study implications

Use of garbage codes to describe UCoD can result from a lack of sufficient information to correctly establish the true UCoD (i.e., the certifying doctor does not have enough information on the chain of events leading to death), faults in the certification and coding processes, or a combination thereof [17]. Individuals with lower education usually have a larger number of co-existing conditions (comorbidities) and a more complex clinical situation [18, 19] compared to those with higher education. In such situations, it can be more challenging to correctly identify the true UCoD. This might be especially relevant for deaths occurring in nursing homes, as these institutions accommodate a very frail subpopulation of the elderly with potentially several comorbidities. For deaths occurring at home, a potential explanation for the observed educational gradients in the use of HF listed as UCoD may relate to the fact that individuals with low(er) SES more frequently live alone [20]. Hence, we would expect a larger proportion of unattended deaths (i.e., deaths not being witnessed) among them, a factor known to increase the use of garbage codes [21]. The above-described mechanisms are, however, less likely to explain the observed educational gradients among those individuals who died in hospital. In these cases, a full history of disease on arrival, supplied by the relevant examinations, should have been sufficient to obtain a complete overview of the chain of events leading to the deceased’s death, regardless of educational status. It seems more likely that in these cases, the potentially accessible information was not obtained as it should have been, or not documented properly in the death certificates. Qualitative studies and focus groups of the health personnel involved in the process of completing death certificates are needed to better understand the reasons, beyond simple errors, underlying the incorrect completion of death certificates.

Lastly, in the context of educational disparities in cause-specific mortality, one might argue that within each ICD chapter—representing broad categories of causes of death—the proportion, structure, and significance of the garbage codes could vary. Consequently, the observed differences in the use of HF codes across educational strata may be partially influenced by variations in cause-specific mortality. To address this, we focused specifically on deaths classified within the CVD chapter. This focus was particularly relevant, as CVD represents the leading cause of death and includes the most critical underlying causes—such as ischemic heart disease, cardiomyopathies, and hypertensive heart disease—where HF may have been incorrectly listed as the UCoD. However, this approach cannot entirely exclude the potential influence of UCoD on the extent of garbage code usage.

Regardless of the reasons, our findings point to several implications. First, a greater use of garbage codes among less educated individuals may hinder our understanding of the true drivers of disease and related risk factors in this “marginalized” subpopulation that already has higher morbidity and mortality rates. Second, educational gradients in the quality of mortality data could potentially bias the results of studies focusing on educational gradients in cause-specific mortality (i.e., due to CVD, cancer, and other relevant causes), as differential use of garbage codes across educational strata has the potential to distort the true picture of cause-specific mortality. Third, our study findings indicate that educational gradients—known to influence the occurrence and outcomes of many diseases [6–9]—also extend to the quality of vital statistics.

### Strengths and weaknesses of the study

This is the first nationwide study applying individual-level linkages that has demonstrated an inverse, dose-response association between education level and HF listed incorrectly as UCoD. The study spanned 30 years and included the entire population, with no restrictions on age, sex, or region of residence. Our findings were consistent after adjusting for several factors known to influence the use of garbage codes [15]. Stratified analyses by study period, sex, age, and place of death, as well as restricting analyses to CVD deaths, added to the value of our study.

When interpreting our results, one needs to keep in mind some limitations characterizing registry-based studies. These registries do not include dynamic information on individuals’ lifestyles nor detailed information on medical or social aspects of the final period of an individual’s life. Further, no information is provided on how and by whom the death certificate is completed nor about their experience filling in death certificates. Lastly, our study focused on HF listed as UCoD. More studies focusing on all garbage codes are needed to confirm the existence of educational gradients in the quality of vital statistics.

## Conclusions

We observed a consistent, inverse, dose-response association between education and the use of HF to describe UCoD. More studies, including qualitative and focus group analyses, are needed to understand the mechanisms underlying the suboptimal quality of vital statistics and how these factors may work disproportionally across education strata. To conclude, parallel to the overall efforts to reduce the use of garbage codes, we need to ensure that all SES groups profit equally from these efforts.
